# REP1 inhibits FOXO3-mediated apoptosis to promote cancer cell survival

**DOI:** 10.1038/cddis.2016.462

**Published:** 2017-01-05

**Authors:** Kwon-Ho Song, Seon Rang Woo, Joon-Yong Chung, Hyo-Jung Lee, Se Jin Oh, Soon-Oh Hong, Jaegal Shim, Yong Nyun Kim, Seung Bae Rho, Seung-Mo Hong, Hanbyoul Cho, Masahiko Hibi, Dong-Jun Bae, Sang-Yeob Kim, Min Gyu Kim, Tae Woo Kim, Young-Ki Bae

**Affiliations:** 1 Laboratory of Tumor Immunology, Department of Biomedical Sciences, Graduate School of Medicine, Korea University, Seoul, Republic of Korea; 2 Department of Biochemistry, Korea University College of Medicine, Seoul, Republic of Korea; 3 Department of Biomedical Sciences, College of Medicine, Korea University, Seoul, Republic of Korea; 4 Translational Research Institute for Incurable Diseases, Korea University College of Medicine, Seoul, Republic of Korea; 5 Experimental Pathology Laboratory, Laboratory of Pathology, Center for Cancer Research, National Cancer Institute, National Institutes of Health, Bethesda, MD, USA; 6 Comparative Biomedicine Research Branch, Research Institute, National Cancer Center, Goyang, Republic of Korea; 7 Gynecologic Cancer Branch, Research Institute, National Cancer Center, Goyang, Republic of Korea; 8 Department of Pathology, Asan Medical Center, University of Ulsan College of Medicine, Seoul 05505, Republic of Korea; 9 Department of Obstetrics and Gynecology, Gangnam Severance Hospital, Yonsei University College of Medicine, Seoul, Republic of Korea; 10 Bioscience and Biotechnology Center, Nagoya University, Nagoya, Japan; 11 ASAN Institute for Life Sciences, ASAN Medical Center, Seoul, Republic of Korea; 12 Department of Convergence Medicine, University of Ulsan College of Medicine, Seoul, Republic of Korea; 13 School of Medicine, The Catholic University of Korea, Seoul, Republic of Korea

## Abstract

Rab escort protein 1 (REP1) is a component of Rab geranyl-geranyl transferase 2 complex. Mutations in REP1 cause a disease called choroideremia (CHM), which is an X-linked eye disease. Although it is postulated that REP1 has functions in cell survival or death of various tissues in addition to the eye, how REP1 functions in normal and cancer cells remains to be elucidated. Here, we demonstrated that REP1 is required for the survival of intestinal cells in addition to eyes or a variety of cells in zebrafish, and also has important roles in tumorigenesis. Notably, REP1 is highly expressed in colon cancer tissues and cell lines, and silencing of REP1 sensitizes colon cancer cells to serum starvation- and 5-FU-induced apoptosis. In an effort to elucidate the molecular mechanisms underlying REP1-mediated cell survival under those stress conditions, we identified FOXO3 as a binding partner of REP1 using a yeast two-hybrid (Y2H) assay system, and we demonstrated that REP1 blocked the nuclear trans-localization of FOXO3 through physically interacting with FOXO3, thereby suppressing FOXO3-mediated apoptosis. Importantly, the inhibition of REP1 combined with 5-FU treatment could lead to significant retarded tumor growth in a xenograft tumor model of human cancer cells. Thus, our results suggest that REP1 could be a new therapeutic target in combination treatment for colon cancer patients.

Forkhead box transcription factor class O (FOXO) proteins are important regulators that participate in a variety of cellular processes including cell cycle progression, programmed cell death, stress detoxification, DNA damage repair, glucose metabolism, and differentiation.^1, 2^ In mammals, this Forkhead subfamily consists of four members, of which the three predominant members, FOXO1 (also known as FKHR), FOXO3 (also known as FKHRL1) and FOXO4 (also known as AFX), display a high degree of redundancy in function.^3, 4^ In cancer, FOXOs are considered as tumor suppressor genes because combined somatic deletion of the subfamily causes a progressive cancer-prone condition.^5, 6, 7^ FOXOs participate in the processes of apoptosis and cell cycle arrest by regulating the transcription of genes involved in apoptosis, cell cycle regulation and DNA damage repair.^8^ Specifically, the transcriptional functions and subcellular localization of FOXOs are regulated in part by PI3K/Akt signaling which phosphorylates FOXOs to promote interaction with 14-3-3 protein, resulting in nuclear export and ubiquitin proteasome pathway-dependent degradation of FoxOs.^9, 10^ Of these, FOXO3 is highly expressed in normal tissue, while it is either reduced or restricted to the cytoplasm in tumor tissues.^6, 11, 12^ Collectively, inactivation of FOXOs appears to be a crucial stage in tumorigenesis; hence, restoring the activity of these factors could be a potential effective therapeutic strategy. In addition, modulation of subcellular translocation of FOXOs could provide another possible strategy.

Rab escort protein 1 (REP1) is a cofactor of Rab geranyl-geranyl transferase 2 (GGTase 2), which functions in geranyl-geranyl modification of C-terminal cysteine residues of newborn Rab GTPases that are essential for regulating vesicle trafficking.^13, 14^ Mutations in REP1 in humans cause a disease called choroideremia (CHM) which is an X-linked eye disease characterized by progressive degeneration of retinal pigment epithelium, photoreceptors, and choroid.^15, 16^ Meanwhile, in mammals, there is an additional REP1-like protein, REP2, which may partially compensate the function of REP1 in most of tissues except eyes, thereby CHM phenotype is mainly restricted in eyes.^17, 18^ The functional study of REP1 using animal models also showed that the mutation of the REP1 gene causes defects in photoreceptors and retinal pigment epithelium accompanied by reduction in the number of melanosomes in mice,^19, 20^ and leads to destruction of hair cells and photoreceptor degeneration in zebrafish.^21, 22^ Apart from the characteristic eye degeneration phenotype, the knockout of REP1 led to abnormal trophoblast development and vascularization in extra-embryonic tissues in mice,^23^ and uninflated swim bladders and edema of the heart and abdomen were observed in *rep1* mutant zebrafish.^18^ Thus, it is supposed that REP1 has functions in cell survival or death of various tissues in addition to eyes; however, how the functions of REP1 are controlled in normal and cancer cells remains to be elucidated.

In the present study, we verified that REP1 has important roles in normal development of intestinal cells in zebrafish in addition to eyes, and demonstrated that REP1 function in tumorigenesis, especially colon cancer cell survival under serum starvation- or 5-FU-mediated stress conditions. Furthermore, we show herein novel insights into the roles of REP1 in FOXO3-mediated apoptosis under stress conditions.

## Results

### Cell survival was impaired in the intestine of *rep1*^*−/−*^ mutant zebrafish embryos

**Figure 1:**
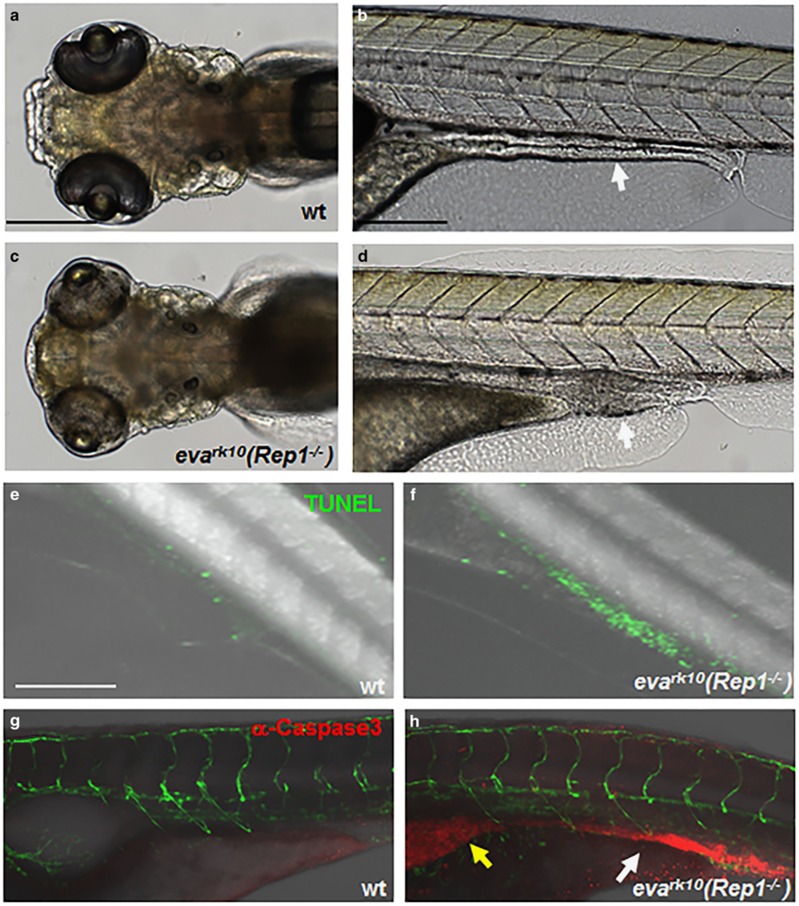
Cell survival was impaired in the intestine of *rep1*^−/−^ mutant zebrafish embryos. (**a** and **c**) Dorsal view images of wild-type zebrafish (**a**) and *rep1*^−/−^ mutant zebrafish embryos (**c**). Lateral view images of wild-type (**b**) and *rep1*^−/−^ mutant zebrafish embryos (**d**) at 5 days post fertilization (dpf). (**d**) Defective eye morphology was detected in *rep1*^−/−^ mutant embryos. The length of the intestine of *rep1*^−/−^ mutant embryo was shortened and it was malformed (white arrow in **d**). (**e** and **f**) TUNEL assays with wild-type (**e**) and *Rep1*^−/−^ mutant embryos (**f**). Extensive TUNEL labeling was observed in the intestine of *rep1*^−/−^ mutant embryos. (**g** and **h**) Immunostaining with anti-caspase-3 in wild-type *Tg(flk:gfp)* (**g**) and *rep1*^−/−^ mutant *Tg(flk:gfp)* embryos (**h**). Apoptotic cell death was detected in the intestine of *rep1*^−/−^ mutant *Tg(flk::egfp)* embryos (red fluorescence indicated with white arrow in **h**) as well as in the stomach of these embryos (yellow arrow in **h**). Blood vessels were visualized with green fluorescence (**g** and **h**). All images were lateral views except for (**a**) and (**c**) at 5 dpf. Scale bar, 200 *μ*m

The mutant for zebrafish *rab escort protein 1 (rep1)* gene was originally screened as the *evanescence (eva*^*rk10*^) mutant that displayed loss of granular cells and reduction of Purkinje cells in the cerebellum.^24^ Positional cloning identified that the *evanescence* mutant phenotype was caused by the mutation of the *rep1* gene, and the truncated form of mutant REP1 protein does not have normal function (unpublished data). The major morphological changes of *eva*^*rk10*^ mutant were small, under-pigmented eyes, similar to those in the previously reported alleles of *rep1* mutants (Figure 1c).^21, 22^ In addition to eye defects, we found that the length of intestine was shortened and it was malformed in mutants compared with wild-type embryos at 5 days post fertilization (dpf) (Figure 1b and d). To examine whether the malformed the intestine in mutants could be due to cell survival defects, we counted the number of TUNEL-positive apoptotic cells in intestines of normal and mutant zebrafish. The number of apoptotic cells was dramatically increased in intestine of *rep1* mutants; meanwhile, apoptotic cells were merely detected in the intestine and other tissues in the zebrafish trunk of wild-type embryos (Figure 1e and f). The increase of apoptotic cell death in intestine of *rep1* mutant embryos was further confirmed by immunostaining with anti-caspase-3 antibody. Consistently, there were very few red fluorescent-positive apoptotic cells in the intestine of wild-type embryos (Figure 1g). In contrast, numerous activated caspase-3-positive apoptotic cells were detected in the intestine as well as the stomach of *rep1* mutant embryos without any defects in blood vessel structures marked by green fluorescence (Figure 1h). These data imply that the normal function of *rep1* gene is necessary for intestine cell survival in the zebrafish embryos.

### Silencing of REP1 sensitizes colon cancer cells to serum starvation- and 5-FU-induced apoptosis

**Figure 2:**
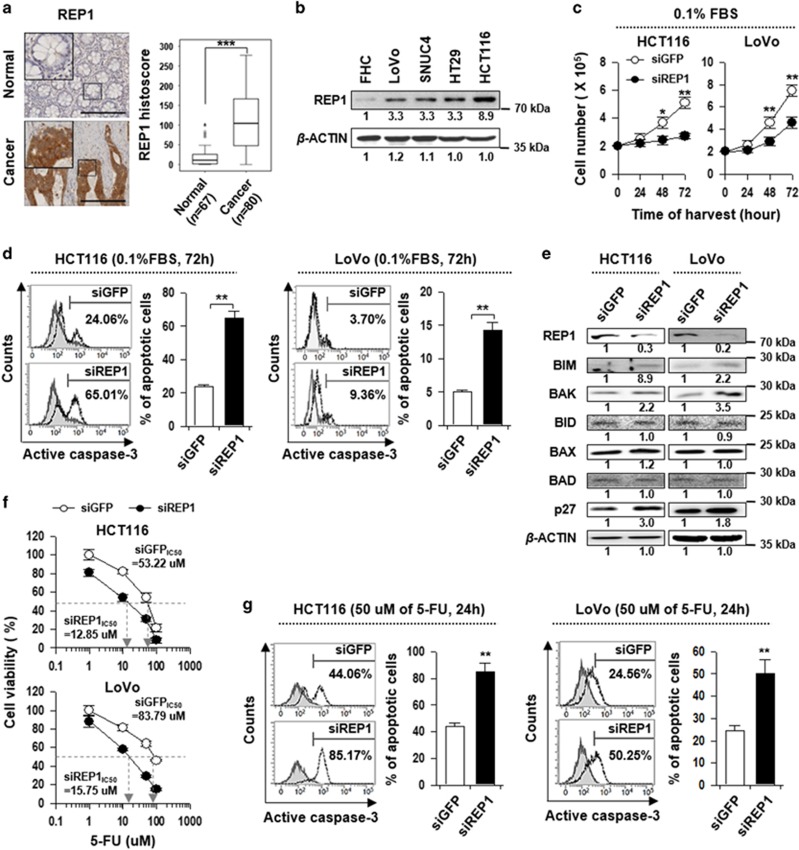
Silencing of REP1 sensitizes colon cancer cells to serum starvation- and 5-FU-induced apoptosis. (**a**) Immunohistochemical staining of REP1 was performed on tissue microarray of human colon cancer specimens. Representative images of immunohistochemical staining of REP1 in colon tissues from normal and carcinoma patients. High-magnification images are shown in inset. Scale bar, 100 *μ*m. (**b**) Protein expression of REP1 in normal colon cells (FHC) and colon cancer cells (LoVo, SNUC4, HT29, HCT116) was determined by immunoblotting. *β*-ACTIN was included as an internal loading control. Numbers below blots indicate the expression as measured by fold change. (**c**–**e**) HCT116 and LoVo cells were transfected with siRNAs targeting GFP or REP1. (**c**) Growth rate of siGFP- *versus* siREP1-transfected HCT116 and LoVo cells in serum starvation culture (0.1% FBS). Cells were harvested at the indicated times and counted after trypan blue staining to exclude dead cells. (**d**) Flow cytometry analysis of the frequency of apoptotic (active caspase-3+) cells among siGFP- or siREP1-transfected HCT116 and LoVo cells. Isotype control staining is indicated by solid gray regions and anti-active caspase-3 staining is indicated by black dotted-lines. (**e**) Expressions of REP1, BIM, BAK, BID, BAX, BAD, and p27 were analyzed by immunoblotting. *β*-ACTIN was included as an internal loading control. Numbers below blots indicate the expression as measured by fold change. (**f** and **g**) HCT116 and LoVo cells were transfected with siRNAs targeting GFP or REP1. Cells were treated with the indicated concentrations of 5-FU for 24 h. (**f**) Viability of siGFP- or siREP1-transfected HCT116 and LoVo was measured by an MTT assay, and then the concentrations resulting in 50% inhibition of cell viability (IC50 values) were determined. Each experiment was performed in triplicate, and error bars represent S.D. from the mean. (**g**) Flow cytometry analysis of the frequency of apoptotic (active caspase-3+) cells among siGFP- or siREP1-transfected HCT116 and LoVo cells. Isotype control staining is indicated by solid gray regions and anti-active caspase-3 staining is indicated by black dotted-lines. All Graphs represent two independent experiments performed in triplicate. Error bars represent S.D. from the mean. *P<0.01, **P<0.001

As knockout of *rep1*-impaired cell survival in the intestine of zebrafish, we hypothesized that functions of REP1 protein associated with cell survival are not only confined to embryonic cells but they also apply to human cells derived from the intestine. To address it, firstly, we determined REP1 expression levels in normal colon tissues and colon cancer tissues. REP1 proteins were highly expressed in colon cancer tissues compared with the normal colon (*P*<0.0001, Figure 2a). In addition, we determined REP1 expression in normal colon cells (FHC) and colon cancer cells (LoVo, SNUC4, HT29, and HCT116). REP1 was highly expressed in colon cancer cells compared with normal colon cells (Figure 2b). These data suggest that REP1 is prominently expressed in colon cancer cells and it could be involved in cancer progression. Therefore, we further verified REP1 functions in survival of HCT116 and LoVo colon cancer cells. There was no considerable difference in cell survival between REP1-silenced colon cancer cells (siREP1) and controls (siGFP) under normal serum condition (data not shown). However, proliferation of siREP1-treated cells was significantly decreased compared with that in controls in a time-dependent manner under serum-starved condition (Figure 2c). In addition, the apoptotic cells were increased about three-fold in each colon cancer cell line by knockdown of REP1 after 72 h under starvation (Figure 2d) and, consistently, the levels of pro-apoptotic molecules such as BIM and BAK, and CDK inhibitor p27, were also increased (Figure 2e). Furthermore, the siREP1-treated HCT116 and LoVo cells (siREP1, IC50=53.22 and 83.79 uM, respectively) were more susceptible to the anti-tumor effect of 5-fluorouracil (5-FU) than the siGFP-treated HCT116 and LoVo cells (siGFP, IC50=12.85 and 15.75 uM, respectively) (Figure 2f). Notably, the siREP1-transfected colon cancer cells were much more vulnerable to apoptosis induced by 5-FU than siGFP-transfected cells (Figure 2g). Taken together, these data indicate that REP1 is required for survival of colon cancer cells against apoptotic cell death induced by the anti-cancer drug 5-FU as well as in serum starvation.

### REP1 confers resistance to serum starvation- and 5-FU-induced apoptosis

**Figure 3:**
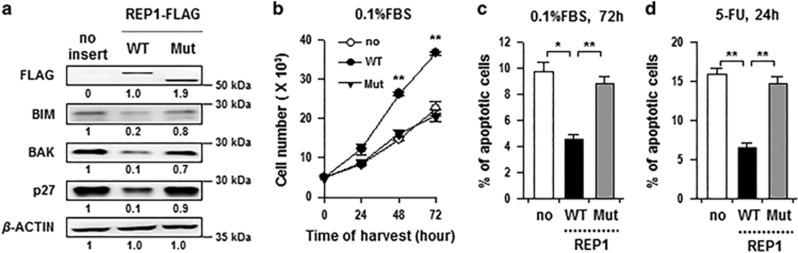
REP1 confers resistance to serum starvation- and 5-FU-induced apoptosis. HEK293 cells were transfected with empty vector (no), REP1 wild-type (WT) or REP1 mutant (Mut). (**a**) Expressions of FLAG (REP1), BIM, BAK, and p27 were analyzed by immunoblotting. *β*-ACTIN was included as an internal loading control. Numbers below blots indicate the expression as measured by fold change. (**b**) Growth rate of transfected cells in serum-starvation culture (0.1% FBS). Cells were harvested at the indicated times and counted after trypan blue staining to exclude dead cells. (**c**) The frequency of apoptotic (active caspase-3+) cells among the transfected cells cultured in serum-starvation condition (0.1% FBS) was estimated by flow cytometry analysis. (**d**) Transfected cells were treated with 50 uM of 5-FU for 24 h and then the frequency of apoptotic (active caspase-3+) cells was determined by flow cytometry analysis. All graphs represent two independent experiments performed in triplicate. Error bars represent S.D. from the mean. *P<0.01, **P<0.001

In addition to loss-of-function studies, we carried out gain-of-function studies for REP1. According to our unpublished data, C-terminal region truncation of the REP1 protein causes the *evanescence* mutant phenotype in zebrafish, suggesting that the mutant form of REP1 does not have the normal function of REP1. To address this, we constructed a mimetic truncated mutant (REP1 Mut) of the zebrafish REP1 mutant, and then, we introduced wild-type REP1 (REP1 WT) and REP1 Mut into HEK293 cells which have low expression of REP1 (Supplementary Figure 1). REP1 WT-transfected cells showed decreased expression of BAK, BIM, and p27 compared with empty vector (no)-transfected cells (Figure 3a). However, transfection of REP1 Mut failed to cause markedly decrease in the expression of BAK, BIM, and p27 (Figure 3a). Transfection of REP1 WT accelerated the growth of HEK293 cells, whereas transfection of REP1 Mut had no substantial impact on cell growth (Figure 3b). Notably, transfection of REP1 WT into HEK293 cells reduced apoptosis induced by serum starvation and 5-FU, while transfection of REP1 Mut did not significantly alter the susceptibility of HEK293 cells to apoptotic stimuli (Figure 3c and d). Collectively, these data indicate that REP1 is able to confer resistance to apoptosis which is induced by serum starvation or anti-tumor drug 5-FU, and the C-terminal region of the REP1 protein is important for the anti-apoptotic function of REP1.

### REP1 physically interacts with FOXO3

**Figure 4:**
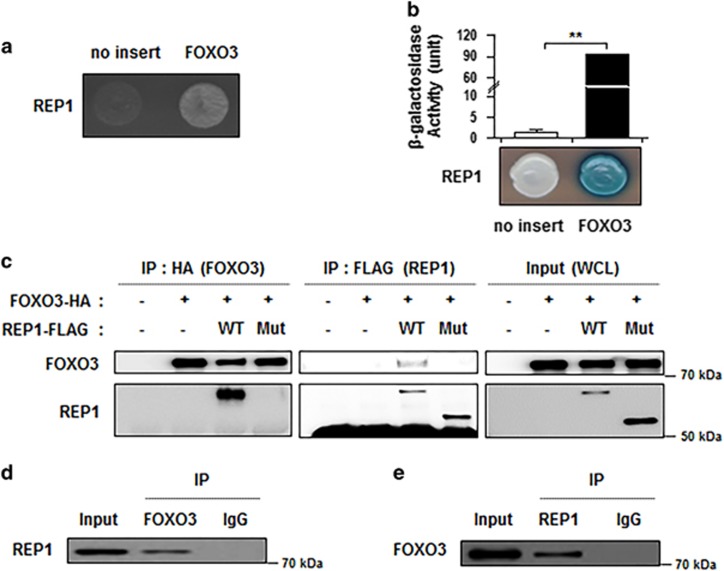
REP1 interacts with FOXO3. (**a** and **b**) Positive protein–protein interactions were determined by monitoring cell growth over 3 days on a medium lacking leucine (**a**), and by the formation of blue colonies on X-gal plates containing galactose at 30 °C (**b**). The values of *β*-galactosidase activity (unit) estimated by adding *o*-nitrophenyl *β*-_D_-galactopyranoside (ONPG) are indicated below their corresponding lanes. Graph represents two independent experiments performed in triplicate. Error bars represent S.D. from the mean. **P<0.001. (**c**) Co-immunoprecipitation of REP1 and FOXO3. HEK293 cells were co-transfected with the indicated constructs. Lysates of the transfected cells immunoprecipitated with anti-HA antibody (left) or anti-FLAG antibody (middle), followed by western blotting using anti-FOXO3 and anti-REP1 antibodies. The input represents 5% of whole-cell lysates (WCL) used in immunoprecipitation (right). (**d**–**e**) Endogenous REP1 binds to endogenous FOXO3 in HCT116 cells. Immunoprecipitation was performed using rabbit IgG, anti-FOXO3 (**d**) or anti-REP1 (**e**), followed by western blotting using the indicated antibodies

Although it has been suggested that REP1 has a crucial role in survival and resistance of colon cancer cells against an anti-cancer drug through conferring anti-apoptotic properties to cells, the underlying mechanism how REP1 confers anti-apoptotic function to cells is largely unknown. To gain insight into the role of REP1 in cell survival and chemo-resistance, we employed a yeast two-hybrid system (Y2H) *in vivo* and screened FOXO3 as a potential REP1-interacting protein. To confirm this result, positive interaction was evaluated using both cell growth on leucine-deficient plates and *o*-nitrophenyl *β*-_D_-galactopyranoside (ONPG) *β*-galactosidase activity. As shown in Figure 4a and b, *β*-galactosidase activity was fully activated (92.22±1.29) for the interaction between REP1 and FOXO3 but it was not activated with the empty-insert plasmid (no insert: 1.38±0.55). To confirm our Y2H results, we carried out co-immunoprecipitation (co-IP) by immunoprecipitation with either anti-HA (FOXO3) or anti-FLAG (REP1) antibody from lysates of HEK293 cells transfected with FOXO3 tagged HA (FOXO3-HA) and REP1-tagged FLAG (REP1-FLAG). Under the IP condition by the anti-HA antibody, only REP1 WT co-precipitated with HA-FOXO3, whereas REP1 Mut did not co-precipitate with HA-FOXO3 (Figure 4c, left). Likewise, under IP using anti-FLAG, only REP1 WT co-precipitated with FOXO3 (Figure 4c, middle). Moreover, endogenous REP1 could indeed co-precipitate with FOXO3, and reciprocal IP using anti-REP1 antibody also pulled down FOXO3 (Figure 4d and e). These findings suggest that REP1 physically interacts with FOXO3 and the C-terminal region of the REP1 protein is required for the interaction.

### siREP1-induced cell death was alleviated by inhibition of FOXO3 in colon cancer cells

**Figure 5:**
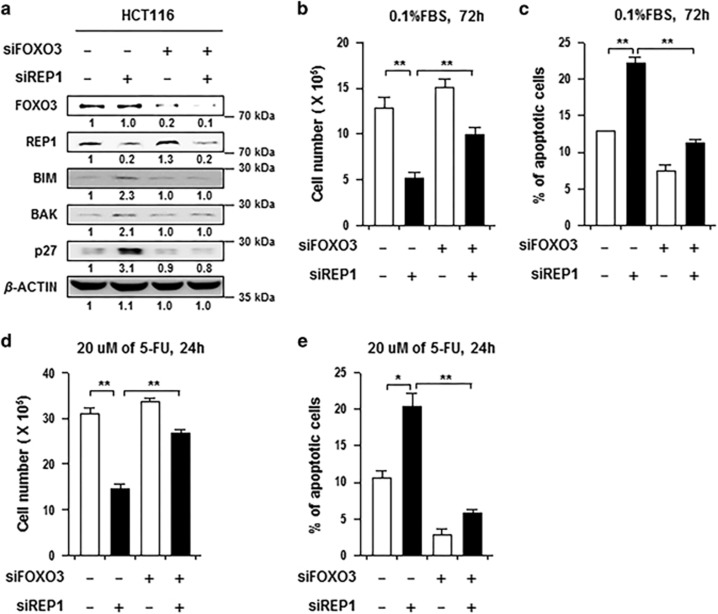
siREP1-mediated cell death was alleviated with inhibition of FOXO3 in colon cancer cells. (**a**–**e**) HCT116 cells were transfected with siRNAs targeting REP1 and FOXO3 as indicated. (**a**) Expressions of FOXO3, REP1, BIM, BAK, and p27 were analyzed by immunoblotting. *β*-ACTIN was included as an internal loading control. Numbers below blots indicate the expression as measured by fold change. (**b** and **c**) Transfected cells were cultured in serum-starvation condition (0.1% FBS). (**b**) Number of live cells was counted by trypan blue staining. (**c**) The frequency of apoptotic cells (active caspase-3+) was determined by flow cytometry analysis. (**d**–**e**) Transfected cells were treated with 20 uM of 5-FU for 24 h. (**d**) Number of live cells was counted by trypan blue staining. (**e**) The frequency of apoptotic (active caspase-3+) cells was determined by flow cytometry analysis. All graphs represent two independent experiments performed in triplicate. Error bars represent S.D. from the mean. *P<0.01, **P<0.001

As FOXO3 is a crucial regulator of apoptosis induced by various stress stimuli or anti-cancer drugs, we next examined whether FOXO3 is required for siREP1-mediated apoptosis under serum-starved conditions and 5-FU treatment. To test this possibility, HCT116 cells were transfected with siGFP or siREP1, together with or without siFOXO3. As observed in Figure 2e, when REP1 was knocked down alone, the expression level of BIM, BAK, and p27 was increased, respectively. Interestingly, even when REP1 was knocked down, the expressions of BIM, BAK, and p27 were not affected if FOXO3 was knocked down (Figure 5a). To further reproduce these phenomena, we repeated the above experiments under serum-starved condition. Consistently, siREP1-transfected cells were less resistant to serum-starved condition and apoptosis than siGFP-transfected cells (Figure 5b and c). However, when we knocked down REP1 and FOXO3 together, we observed a higher number of viable cells and a lower number of apoptotic cells than in REP1 knockdown alone cells (Figure 5b and c). In addition, we also performed cell viability assay under an apoptotic condition induced by 5-FU. Similarly, the negative effect of REP1 knockdown on cell survival was reduced when FOXO3 was knocked down simultaneously (Figure 5d and e). All of these data led us to the conclusion that siREP1-mediated cell death was alleviated by inhibition of FOXO3, that is, REP1 could function in cell survival under various stress conditions by conferring negative effects to FOXO3-dependent apoptosis in colon cancer cells.

### REP1 negatively regulates the transcriptional activity of FOXO3 and the expression of FOXO3 target genes

**Figure 6:**
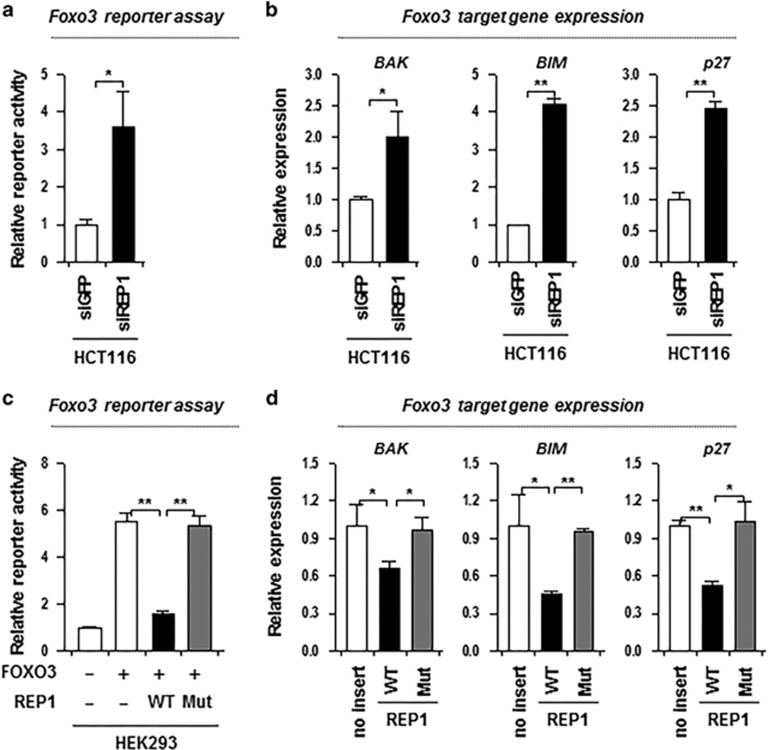
REP1 negatively regulates the transcriptional activity of FOXO3 and the expression of FOXO3 target genes. (**a** and **b**) HCT116 cells were transfected with siRNAs targeting GFP or REP1. (**a**) FOXO3 transcriptional activity was determined using the FHRE-Luc reporter system. The cells were transfected with the FHRE-Luc reporter. A vector expressing *β*-galactosidase was co-transfected to ensure transfection efficiency and to normalize luciferase activity values. For analysis of the FOXO3 reporter activity in the cells, it was normalized to the control cells transfected with the empty vector. (**b**) mRNA expression of BAK, BIM, and p27 was analyzed by real-time quantitative RT-PCR. (**c**) FOXO3 transcriptional activity was determined using the FHRE-Luc reporter system. HEK293 cells were transfected with the FHRE-Luc reporter, together with the indicated plasmids. (**d**) HEK293 cells were transfected with empty vector (no), REP1 wild-type (WT), or REP1 mutant (Mut). mRNA expression of BAK, BIM, and p27 was analyzed by real-time quantitative RT-PCR. Fold change was calculated relative to the expression level of mRNA in control cells. All graphs represent two independent experiments performed in triplicate. Error bars represent standard deviations from the mean. *P<0.01, **P<0.001

Transcription factor FOXO3 controls cell death *via* upregulating transcription of genes related to apoptosis or cell cycle arrest such as BIM, BAK, and p27.^25^ To confirm that REP1 inhibits FOXO3 function, we used the FHRE-Luc reporter system, which expresses luciferase when FOXO binds to FHRE. To assess the transcriptional activity of FOXO3 in the presence of REP1, HCT116 cells were transfected with siGFP or siREP1. Relative reporter activity in siREP1 group was significantly increased, compared with that in siGFP (Figure 6a). Notably, the expression of FOXO3 target genes such as BAK, BIM, and p27 was increased when REP1 was knocked down (Figure 6b). To confirm the inhibitory effect of REP1 on FOXO3 transcriptional activity, we overexpressed the wild-type and mutant REP1 in HEK293 cells, and then analyzed the transcriptional activity of FOXO3 with FHRE-Luc. Consistently, when HEK293 cells were transfected with wild-type REP1, FOXO3-mediated reporter activity was significantly reduced (Figure 6c). However, mutated REP1 did not inhibit FOXO3-mediated reporter activity (Figure 6c). Also, the transcription level of all three FOXO3 target genes was decreased when wild-type REP1 was overexpressed, whereas mutant REP1-overexpressing cells did not show a remarkable difference from no insert (Figure 6d). From these data, we concluded that REP1 negatively regulates the transcriptional activity of FOXO3 and the expression of FOXO3 target genes, including BAK, BIM, and p27.

### REP1 inhibits nuclear trans-localization of FOXO3

**Figure 7:**
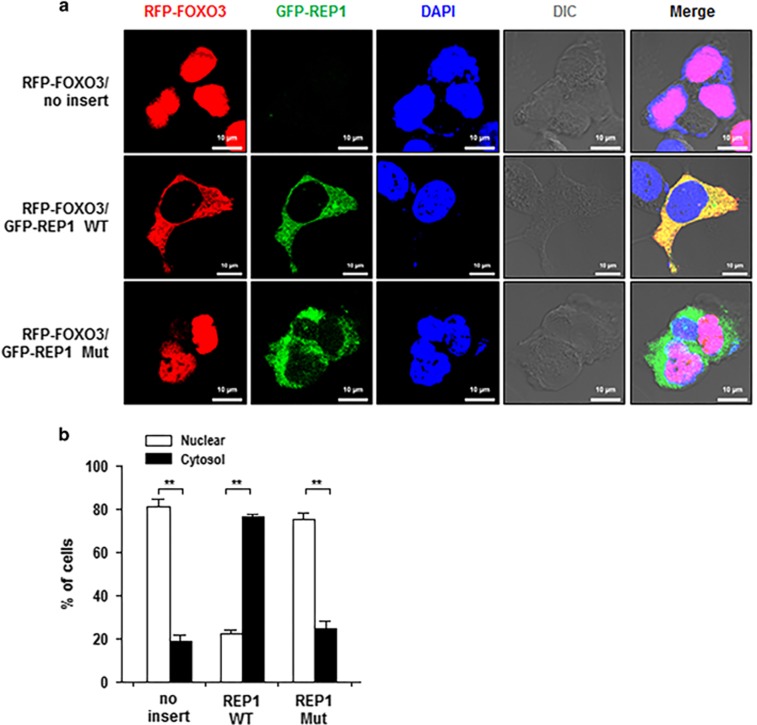
REP1 inhibits nuclear trans-localization of FOXO3. HEK293 cells were transfected with RFP-FOXO3 together with empty vector (no), GFP-REP1 WT, or GFP-REP1 Mut. Confocal fluorescent microscopy was used to evaluate the subcellular distribution of FOXO3 and REP1. Representative pictures are shown in (**a**), and the experimental quantitation of subcellular localization of FOXO3 is shown in (**b**). Graph represents three independent experiments and error bars represent S.D. from the mean. **P<0.001

Being a transcription factor, FOXO3 activity is highly influenced by its subcellular localization. Since REP1 silencing did not alter the expression of FOXO3 (see Figure 5a), we reasoned that the inhibitory function of REP1 on transcriptional activity of FOXO3 might be associated with trans-localization of FOXO3. To test this possibility, we examined the subcellular localization of FOXO3 depending upon REP1 expression by transfecting RFP-FOXO3 plasmid into HEK293 cells. When we transfected the empty vector (no insert), RFP-FOXO3 proteins were mainly localized within the nucleus rather than the cytoplasm. However, RFP-FOXO3 proteins were definitely localized in the cytoplasm of HEK293 cells which were transfected with REP1 WT, while REP1 Mut did not affect the localization of FOXO3 (Figure 7). Furthermore, the majority of RFP-FOXO3 proteins in siGFP-transfected HCT116 cells were localized in the cytoplasm. However, in the absence of REP1, RFP-FOXO3 proteins re-localized themselves within the nucleus (Supplementary Figure 2). From all these data, we could finally conclude that REP1 directly binds to FOXO3 proteins and holds back their re-localization from the cytoplasm to the nucleus.

### Inhibition of REP1 enhances the anti-tumor effect of 5-FU

**Figure 8:**
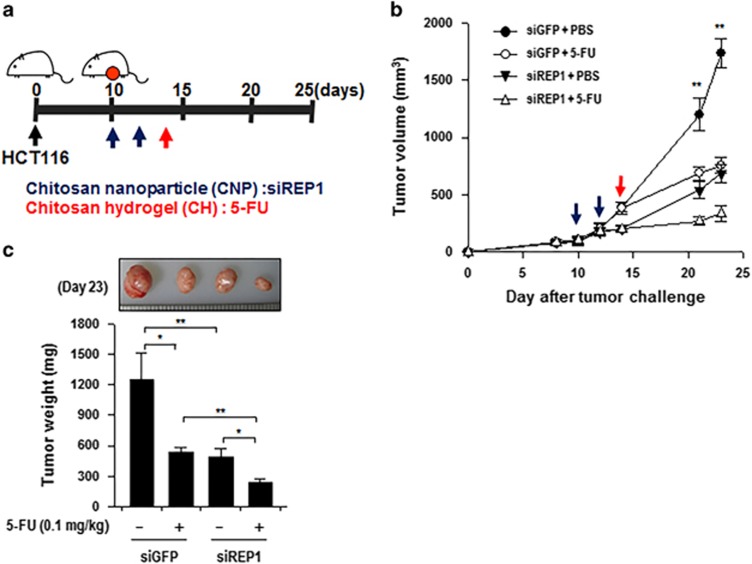
Inhibition of REP1 enhances the anti-tumor effect of 5-FU. (**a**) Schematic representation of the therapy regimen in mice implanted with HCT116 colon cancer cells. Nude mice were inoculated subcutaneously with 1 × 10^6^ HCT116 cells per mouse. Ten days following tumor challenge, siRNA CNP targeting GFP or REP1 (7 ug/mouse) was injected intravenously, twice for 2 consecutive days. Chitosan hydrogels loaded with 5-FU (0.1 mg/kg) were administered intratumorally at day 14. (**b**) Tumor growth in mice inoculated with HCT116 treated with the indicated reagents (five mice/group). (**c**) Tumor mass in mice at 23 days after challenge. Error bars represent S.D. from the mean. *P<0.01, **P<0.001

Because REP1 expression confers resistance to 5-FU *in vitro*, we reasoned that inhibition of REP1 may serve as an interventional strategy for 5-FU-based cancer therapy. To test the therapeutic value of REP1 inhibition, we inoculated HCT116 cells into nude mice, and 10 days later, we knocked down the REP1 expression by intravenous administration of chitosan nanoparticles (CNP) carrying REP1- or GFP-targeting siRNA, according to the schedule described in Figure 8a. CNPs have been previously demonstrated to be highly efficient in delivering siRNA into the tumor mass via the enhanced permeability and retention effect.^26, 27^ Fourteen days after tumor challenge, chitosan hydrogels (CHs) loaded with 5-FU (0.1 mg/kg) were administered intratumorally (Figure 8a). Delivery of siREP1 alone elicited a profound therapeutic effect, and, more importantly, when combined with 5-FU treatment, the tumor burden was significantly reduced compared with siREP1 alone or 5-FU treatment combined with control siRNA at 23 days after tumor challenge (Figure 8b and c). Taken together, our data demonstrate that inhibition of REP1 represents a promising strategy for anti-cancer therapy.

## Discussion

According to previous reports, a loss of function mutation of the REP1 has been known to mainly induce eye disease, CHM, an X-linked recessive disease of the retina that results in progressive degeneration of the retina, RPE, and choroid in mammal.^15, 21, 28, 29^ However, some reports showed that the inactivation of *REP1* gene led to multiple defects in the cerebellum or hair cells of zebrafish,^21, 24^ abnormal trophoblast development and vascularization in mouse extra-embryonic tissues,^23^ and aberrant intracellular transport in fibroblast and monocyte of CHM patients.^29^ In this study, we verified that REP1 has an important role in survival of intestinal cells in addition to eyes or a variety of tissues in zebrafish. These results essentially consist with previous report that *rep1*^−/−^ zebrafish shows the degenerative phenotypes in retina, followed by multisystem disease, and eventually died at an average of 4.8 dpf when the maternally derived REP1 in the yolk sac is exhausted.^18^ Seabra and colleagues additionally showed that the early embryonic lethality was explained by a single *rep1* gene in zebrafish.^18^

Intriguingly, the expression level of REP1 is upregulated in human colon cancer tissues and cell lines compared with the normal counterparts. These data present the possibility that REP1 may function in tumorigenesis, especially cancer cell survival in colon cancer. Overexpression of REP1 in HEK293 cells enhanced cell proliferation and reduced apoptosis in serum-starved condition or during anti-cancer drug 5-FU treatment, which implies that upregulation of REP1 is closely associated with tumorigenesis as well as chemo-resistance. In the knockdown experiment, we found that REP1 is essential for survival of colon cancer cells in serum-starved condition as well as 5-FU-treated condition. It is interesting to note that there was a little difference in survival of colon cancer cells between control and REP1 silencing under normal serum condition. In general, REP1 has a pivotal role in geranyl-geranyl modification of most of Rab GTPases, which function in vesicle trafficking.^14, 30^ A number of Rab protein has been shown to be involved in various stages of autophagy, which is a highly conserved intracellular and lysosome-dependent degradation process, and induced by various forms of cellular stress including nutrient deprivation, reactive oxygen species (ROS) or even DNA damage.^31^ REP1 also functions in geranyl-geranyl modification of autophagy related Rab proteins such as Rab1, Rab5, Rab7, and Rab11.^32, 33^ It needs to be further studied what Rab proteins cooperate with REP1 in normal and cancer cells. Meanwhile, both in yeast and vertebrates, the cell growth and division are governed by environmental nutrient availability, and the conserved target of rapamycin (TOR) signaling mediates the nutrient status to critical cellular processes including ribosome biogenesis, autophagy, and cell cycle entry into G0.^34, 35^ Interestingly, in yeast, *MRS6*, a yeast homolog of vertebrate *choroideremia* (*chm*, *REP1*) gene, functions in integrating the secretion system with TOR signaling and ribosome biogenesis in response to extracellular nutrient level, and suggested as a positive regulator of TORC1 through nuclear translocation of Sfp1 which is downstream effector of TOR signaling.^36, 37^ Hence, it is possible that REP1 may regulate cancer cell survival in serum-starved condition by vesicle trafficking independent manners as well as traditional vesicle trafficking dependent manner.

In the course of elucidating how REP1 functions in survival of colon cancer cells, we identified FOXO3 as a physical interactor for REP1 by Y2H (Figure 3). FOXO3 is a member of Forkhead box O (FoxO) transcription factors which are involved in the regulation of metabolism, lifespan, cell cycle, and apoptosis; moreover, in model organisms, their activity also affects age-related diseases, such as diabetes and cancer.^38^ Especially in cancer, FOXOs inhibit cell proliferation and induce the expression of pro-apoptotic genes or death ligands,^39, 40, 41^ and primarily function in response to environmental changes, including growth factor deprivation, metabolic stress (starvation) and oxidative stress.^42, 43, 44^ In this study, we demonstrated that REP1 functions in colon cell survival depending on the activity of FOXO3 under serum-starved or 5-FU induced oxidative stress conditions (Figure 5), by which REP1 negatively regulates the transcriptional activity of FOXO3 and the expression of FOXO3 target genes, including BAK, BIM, and p27 (Figure 6).

Since it is well known that the transcriptional activity of FOXOs is regulated through post-translational modifications, including phosphorylation, acetylation, methylation and ubiquitination, and/or coordinated shuttling of both FOXOs and their regulators,^38^ we also examined the nuclear-cytoplasmic localization of REP1 and FOXO3. Our observations that REP1 directly binds to FOXO3 proteins, and REP1-dependent cytoplasmic re-localization of FOXO3 is closely correlated with survival of colon cancer cells, are quite similar to the previously proposed mechanisms which show that nuclear-cytoplasmic shuttling of FOXOs is regulated by Akt and 14-3-3.^45, 46^ Taken together, these data indicate that REP1 is a novel regulator for nuclear-cytoplasmic shuttling of FOXO3, during which it regulates cell survival under stress or starvation condition.

On the basis these new findings that REP1 confers resistance to 5-FU which is first-line drug of colorectal cancers. We believe that inhibition of REP1 may be an effective strategy for 5-FU-based cancer therapy. In this study, we provided proof of this concept by knocking down REP1 in a xenograft mouse model of human colon cancer. Notably, in this setting, REP1 inhibition alone strongly retarded tumor growth, and when it was combined with 5-FU treatment, it synergized the anti-tumor effects. Our results indicate that REP1 inhibition is employed as a sensitizer to enhance therapeutic effects of 5-FU and suggest that REP1 could be a new therapeutic target in combination treatment for colon cancer patients.

In summary, we demonstrated that REP1 is required for the survival of intestinal cells in addition to eyes or a variety of cells in zebrafish, and presented that REP1 also has important roles in tumorigenesis, especially colon cancer cell survival under stress conditions including nutrient starvation or 5-FU-mediated stress conditions. We identified FOXO3 as a REP1 interacting protein by Y2H, and found that REP1 inhibits nuclear trans-localization of FOXO3 through physically interacting with FOXO3, thereby inhibiting FOXO3-mediated apoptosis. Furthermore, we showed that REP1 silencing combined with 5-FU treatment could lead to a synergic retardation of tumor growth in a xenograft tumor model of human cancer cells. Therefore, our data provide a new clinical insight for treating colon cancers by regulating FOXO3 with REP1, a novel oncogenic protein.

## Materials and methods

### Zebrafish wild-type, mutant and transgenic lines, maintenance, and immunostaining

Wild-type zebrafish used in this study had the Oregon AB genetic background. The *evanescence (eva* ^*rk10*^) mutant which is the mutant for *rab escort protein 1* (*rep1*)^24^ and *Tg(flk:gfp)*^*s843*^ (ref. 47) was maintained in accordance with the accepted standard procedures^48^ approved by the IACUC at National Cancer Center in Korea (permit number: NCC-15-116C). For whole mount staining, zebrafish embryos were incubated with 0.006% of phenylthiourea (P7629, Sigma, St. Louis, MO, USA) at 12 hpf (hours post fertilization) to prevent pigmentation until 5 dpf (days post fertilization). After fixation in 4% paraformaldehyde overnight at 4 °C, embryos were washed three times with PBS, followed by 100% methanol immersion at −20 °C. For TUNEL assays, embryos were rehydrated progressively (75%, 50%, 25%, and 0% of methanol in PBS), treated with proteinase K (10 mg/ml, for 30 min at room temperature). The apoptotic cells in embryos were detected with the DeadEnd Colorimetric TUNEL System (G7360, Promega, Madison, WI, USA) according to the manufacturer's instructions. For immunostaining, anti-phospho p44/42 MAPK (dp-ERK, 1/200 dilution, Rabbit monoclonal, #4370, Cell Signaling, Danvers, MA, USA) and anti-cleaved caspase-3 (Asp175, 1/200, Rabbit monoclonal, #9661, Cell Signaling) antibodies were used. The procedure for immunostaining was essentially the same as that in a previous report.^24^ The secondary antibody used for visualization with fluorescence was Alexa Fluor 488 or 555 goat-anti-rabbit antibody (1/400 dilution, Molecular Probes, Invitrogen, Carlsbad, CA, USA). After three washes with PBST, the samples were embedded in 1% low-melting-point agarose dissolved in PBS in a glass-bottomed dish, and subjected to confocal imaging (Carl Zeiss LSM510 with CCD camera; Carl Zeiss, Oberkochen, German).

### Immunohistochemistry

Tissue microarrays (TMAs) were constructed from formalin-fixed paraffin-embedded tissue blocks of 80 surgically resected colon cancers with a manual tissue microarrayer (UniTMA Co Ltd, Seoul, Korea). TMAs contained four 2.0 mm cores from three primary colon cancers and matched nonadjacent normal colonic mucosa. The TMA sections were baked at 60 °C for 30 min and then deparaffinized with xylene and dehydrated through a graded ethanol series. Antigen retrieval was achieved for 20 min in heat-activated antigen retrieval pH 6.0 (Dako, Carpinteria, CA, USA) using a pressure cooker (Dako). Endogenous peroxidase activity was quenched with 3% H_2_O_2_ in water for 15 min. The sections were incubated with mouse monoclonal anti-REP1 antibodies (sc-23905, Santa Cruz) at 1:500 for 1 h. Subsequently, antigen–antibody reaction was detected with EnVision+ mouse Link System-HRP (Dako) and visualized with DAB+ (3, 3′-Diaminobenzidine; Dako). Tissue sections were lightly counterstained with hematoxylin and then examined by light microscopy. Negative controls (substitution of primary antibody with TBS) were run simultaneously.

The evaluation of immunostaining was carried out using digital image analyzing software version 4.5.1.324 (Visiopharm, Hoersholm, Denmark). Immunohistochemically stained slides were scanned using NanoZoomer 2.0 HT (Hamamatsu Photonics, Hamamatsu, Japan) at × 20 objective magnification (0.5 *μ*m resolution), and captured digital images were then imported into the Visiopharm software. The intensity of staining was categorized as 0, 1+, 2+, and 3+ according to the distribution pattern across the TMA cores. The final histoscore was calculated by multiplying the intensity and percentage of staining resulting in score of 0–300 as described previously.^49^ Statistic analyses were performed with SPSS version 21.0 for Windows (Chicago, IL, USA). A *P*-value<0.05 was considered statistically significant.

### Mice

Six- to eight-week-old female nude mice were purchased from Central Lab. Animal Inc. (Seoul, Korea). All mice were maintained and handled under the protocol approved by the Korea University Institutional Animal Care and Use Committee (KUIACUC-2014-175). All animal procedures were performed in accordance with recommendations for the proper use and care of laboratory animals.

### Cells

The human colon cancer cells (LoVo, SNUC4, HT29, and HCT116) and human embryonic kidney 293 (HEK 293) cells were purchased from American Type Culture Collection (ATCC, Rockville, MD, USA). HCT116 and HEK293 cells were cultured in DMEM (Thermo Scientific, Waltham, MA, USA) containing 100 units/ml of penicillin-streptomycin and 10% fetal bovine serum (FBS). LoVo, SNUC4, and HT29 cells were cultured in RPMI 1640 containing 100 units/ml of penicillin-streptomycin and 10% FBS. All cells were grown at 37 °C in a 5% CO_2_ incubator/humidified chamber. Normal fetal human colon cell line, FHC, was kindly provided by Dr. JW Hyun (Jeju National University). Human colon FHC cells were cultured in a 1 : 1 mixture of Ham's F12 and DMEM containing HEPES (25 mM), cholera toxin (10 ng/ml; Calbiochem-Novabiochem Corp., La Jolla, CA, USA), insulin (5 *μ*g/ml), transferrin (5 *μ*g/ml), hydrocortisone (100 ng/ml), and 10% FCS.

### DNA constructs

To make the full-length (hREP1-full, 1–653 a.a.) and mutant (hREP1-S473X, 1–472 a.a., which corresponds to the zebrafish mutant protein of *eva*^*rk10*^) human *rep1* expression constructs, we performed PCR from HEK293T cDNA using KOD-Plus-Neo polymerase (TOYOBO Co. Ltd., Osaka, Japan) with the following primers: 5′-CCATCGATTATGGCGGATACTCTCCCTTCG-3′ (5′-primer for hREP1-full and hREP1-S473X) and 5′-GTAGGCGCGCCTTCAGAGGACTCCTCTAGGTT-3′ (3′-primer for hREP1-full), and 5′-TAGGCGCGCCAATCTGTTGACTGAATCTGTTTTTAG-3′ (3′-primer for hREP1-S473X). Each fragment was then inserted into the Cla1 and Asc1 sites of pCS4+ vectors (3xFlag and EGFP).^50^ To make the full-length human FOXO3a for expression in cells, we also performed PCR with the following primers: 5′-AGATCGATATGGCAGAGGCACCGGC-3′ (5′-primer) and 5′-TAGGCGCGCCTGGCACCCAGCTCTGAGA-3′ (3′-primer), and fused it to pCS4+ vectors (3xHA, and mRFP).

### siRNA constructs

Synthetic small interfering RNA (siRNA) specific for GFP, REP1, and FOXO3 were purchased from Bioneer (Daejeon, Korea); Non-specific GFP (green fluorescent protein), 5′-GCAUCAAGGUGAACUUCAA-3′ (sense), 5′-UUGAAGUUCACCUUGAUGC-3′ (antisense), REP1, 5′-CCGGAGAGUUCUGCAUGUU-3′ (sense), 5′-AACAUGCAGAACUCUCCGG-3′ (antisense), FOXO3, 5′-GACGAUGAUGCGCCUCUCU-3′ (sense), 5′-AGAGAGGCGCAUCAUCGUC-3′ (antisense). For *in vitro* delivery, cells were transfected with 100 pmol of synthesized siRNAs using Lipofectamine 2000 (Invitrogen) according to the manufacturer's instructions. For systemic *in vivo* delivery of siRNA into tumor cells, we prepared the characterized CNP as described previously.^51^ Briefly, tripolyphosphate (0.25% w/v) and siRNA (1 ug/ul) were added into the RGD-Chitosan solution, and the siRNA/RGD-CNPs were incubated at 4 °C for 40 min and collected by centrifugation at 13 000 r.p.m. for 40 min at 4 °C. The pellet washed was three times using sterile water. Isolated siRNA/RGD-CH-NP was intravenously injected into HCT116-bearing nude mice.

### Western blot analysis

Lysate extracted from a total of 1 × 10^5^ cells was used to perform western blot as described previously.^43^ Primary antibodies against REP1 (LS-B8700, LSBio, Seattle, WA, USA), Bim (#2819, Cell Signaling), BAK (#556396, BD Biosciences, San Diego, CA, USA), BID (#2002, Cell Signaling), BAX (sc-493, Santa Cruz Biotechnology, Santa Cruz, CA, USA), BAD (#9292, Cell Signaling), p27 (sc-528, Santa Cruz), FLAG (M185-3L, MBL, Nagoya, Japan), FOXO3 (sc-11351, Santa Cruz), HA (M180-3, MBL) and *β*-ACTIN (M177-3) were used in western blotting, followed by the appropriate secondary antibodies conjugated with horseradish peroxidase. Immunoreactive bands were developed using the chemiluminescence ECL detection system (Elpis Biotech, Daejeon, Korea), and signals were detected using a luminescent image analyzer (LAS-4000 Mini, Fujifilm, Tokyo, Japan). *β*-ACTIN was included as an internal loading control. The intensity of the western blot signals was quantified using Multi-gauge software (Fujifilm).

### Flow cytometry analysis

1 × 10^5^ cells were harvested by trypsinization, washed and re-suspended in PBS. To detect apoptotic cells, cells were then labeled with PE-conjugated anti-active caspase-3 antibody (51-68655X, BD Biosciences), according to the manufacturer's instructions. The percentage of apoptotic cells was analyzed using a FACSVerse flow cytometer (BD Biosciences) by determining the active caspase-3^+^ cells. Data acquisition was performed on a FACSVerse flow cytometer with BD FACSuite software.

### Cell viability assay

Cell viability was determined by Trypan blue exclusion assay and MTT assay. For Trypan blue exclusion assay, cells were harvested at the indicated times and stained with 0.4% Trypan blue to exclude dead cells. The number of live cells was counted using a hemocytometer and light microscopy.

### Yeast two-hybrid (Y2H) assay and quantitation of protein–protein interaction

For bait construction with human REP1, cDNA encoding full-length human REP1 was cloned into the *Bam*HI and *Nco*I restriction enzyme sites of the pGilda/LexA yeast shuttle cloning vector. The bait pGilda/LexA-REP1 plasmid was introduced into a yeast strain EGY48 [*MATa, his3, trp1, ura3-52, leu2::pLeu2-LexAop6/pSH18-34* (*LexAop-lacZ* reporter)] using a modified lithium acetate protocol.^52, 53^ Approximately 5.5 × 10^6^ independent transformants were pooled. After re-spreading on selection media (Ura-, His-, Trp-, Leu-) and X-gal-containing plates, and obtaining six positive colonies, the purified plasmids were sequenced. A homology search in the GenBank using the BLAST program identified that the plasmids encoded FOXO3 (accession number: NM_001455.3). The human FOXO3 cDNA encoding a full-length gene was cloned into multi-cloning sites (MCS) of the pJG4-5 plasmid vector, which included B42 fusion proteins (Clontech, Palo Alto, CA, USA). The FOXO3 cDNAs encoding pJG4-5 fusion proteins were transformed into yeast competent cells that already contained pGilda/LexA-REP1, while the transformants were selected based on their tryptophan prototrophy (plasmid vector marker) on a synthetic medium (Ura, His, Trp) containing 2% (w/v) glucose. Positive protein–protein interactions were monitored as the formation of blue colonies on the X-gal-containing medium as described previously.^53^ The binding activity of the interaction was compared by calculating the relative expression level of ONPG *β*-galactosidase.^52^

### Immunoprecipitation

For co-immunoprecipitation of REP1 and FOXO3, HEK293 cells expressing the indicated constructs were cultured for 48 h, and whole-cell lysates were prepared with NP40 lysis buffer (50 mM Tris-HCL, pH 8.0, 5 mM EDTA, 150 mM NaCl, 1% NP-40, 1 mM PMSF) containing protease inhibitor. Immunoprecipitation was carried out by incubation with 1 *μ*g of anti-HA (M180-3, MBL), anti-FLAG (M185-3L, MBL) antibody or mouse IgG for 16 h. For immunoprecipitation of endogenous REP1 or FOXO3, HCT116 cells were lysed in NP40 lysis buffer containing protease inhibitor. Immunoprecipitation was carried out by incubation with 1 *μ*g of anti-REP1 (LS-B8700, LSBio), anti-FOXO3 (sc-11351, Santa Cruz) antibody or rabbit IgG for 16 h. The bound proteins were eluted by boiling in SDS sample buffer and were detected by western blotting.

### Real-time quantitative RT-PCR

Total RNA was isolated using RNeasy Micro kit (Qiagen, Valencia, CA, USA), and the cDNAs were synthesized by reverse transcriptase (RT) using iScript cDNA synthesis kit (Bio-Rad, Hercules, CA, USA), according to the manufacturer's recommended protocol. Real-time quantitative PCR was performed using iQ SYBR Green super mix (Bio-Rad) with the following specific primers; BIM, 5′-AGGCAATCACGGAGGTGAAG-3′ (forward) and 5′-TCTGGTAGCAAAAGGGCCAG-3′ (reverse); BAK, 5′-TCATCGGGGACGACATCAAC-3′ (forward) and 5′-CAAACAGGCTGGTGGCAATC-3′ (reverse); p27, 5′-CAGCTTGCCCGAGTTCTACT-3′ (forward) and 5′-TGTCCTCAGAGTTAGCCGGA-3′ (reverse); *β*-Actin, 5′-CGACAGGATGCAGAAGGAGA-3′ (forward) and 5′-TAGAAGCATTTGCGGTGGAC-3′ (reverse) on a CFX96 real-time PCR detection system. All real-time quantitative PCR experiments were performed in triplicate and quantification cycle (Cq) values were determined using Bio-Rad CFX96 Manager 3.0 software. Relative quantification of the mRNA levels was performed using the comparative Ct method with *β*-Actin as the reference gene.

### Luciferase assay

To determine the transcriptional activity of FOXO3, luciferase assay was performed as described previously,^51^ using FHRE-Luc, which was a gift from Michael Greenberg (Addgene plasmid # 1789).^54^ Briefly, using Lipofectamine 2000 (Invitrogen), cells were transfected with 100 ng of FHRE-Luc reporter, and 100 ng of indicated constructs, together with 20 ng of CMV/*β*-galactosidase plasmid to normalize transfection efficiency. After 24 h, cells were washed with PBS and permeabilized with Cell Culture Lysis Reagent (Promega). Luciferase activity was measured with a Turner Biosystems TD-20/20 luminometer after addition of 40 μl of luciferase assay reagent (Promega). *β*-galactosidase activity was measured with a uQuant microplate reader (BioTek, Winooski, VT, USA) at 570 nm wavelength after addition of *β*-galactosidase assay reagent containing 1 mM chlorophenol red *β*-d-galactopyranoside substrate (Roche).

### Immunofluorescence

To examine cellular localization of FOXO3, cells were cultured on 2-well cell culture slides (#30102, SPL Life Science, Suwon, Korea) and transfected with the indicated plasmid DNAs or siRNAs using Lipofectamine2000 (Invitrogen) according to the manufacturer's protocol. The transfected cells were fixed and permeabilized with Cytofix/Cytoperm (BD Biosciences) for 20 min at 4 °C. After washing in 1 × Perm-wash buffer and nuclear counterstaining with DAPI, localization of FOXO3 or REP1 was demonstrated under a confocal laser scanning microscope (ZEISS LSM700, Carl Zeiss).

### Tumor treatment experiments

Nude mice were inoculated subcutaneously with 1 × 10^6^ HCT116 cells per animal and siGFP- or siREP1-loaded CNPs (7 *μ*g/mouse) were administered via the tail vein at days 10 and 12. Fourteen days after tumor challenge, chitosan hydrogels loaded with 5-FU (0.1 mg/kg) were administered intratumorally. Mice were monitored for tumor burden for 23 days after challenge.
