# Supplementary material for: REP1 inhibits FOXO3-mediated apoptosis to promote cancer cell survival
Source: Cell Death Dis. 2017 Jan 5;8(1):e2536–. doi: 10.1038/cddis.2016.462 (PMC5386371; doi:10.1038/cddis.2016.462)

**SUPPLEMENTARY INFORMATION**

**REP1 inhibits FOXO3-mediated apoptosis to promote cancer cell survival**

Kwon-Ho Song, Seon Rang Woo, Joon-Yong Chung, Hyo-Jung Lee, Se Jin Oh, Soon-Oh Hong, Jaegal Shim, Yong Nyun Kim, Seung Bae Rho, Seung-Mo Hong, Hanbyoul Cho, Masahiko Hibi, Dong-Jun Bae, Sang-Yeob Kim, Min Gyu Kim, Tae Woo Kim and Young-Ki Bae

**Supplementary Figure 1.** REP1 expression in HEK293, LoVo and HCT116 cells.

**Supplementary Figure 2.** REP1 silencing promotes cytoplasmic retention of FOXO3.

**SUPPLEMENTARY FIGURE LEGENDS**

**Supplementary Figure 1.** REP1 expression in HEK293, LoVo and HCT116 cells. Protein expression of REP1 in HEK293, LoVo and HCT116 cells was determined by immunoblotting. *β*-ACTIN was included as an internal loading control. Numbers below blots indicate the expression as measured by fold change.

**Supplementary Figure 2.** REP1 silencing promotes cytoplasmic retention of FOXO3. HCT116 cells were transfected with RFP-FOXO3, after 24 hours of transfection of siRNAs targeting GFP or REP1. Representative pictures are shown in (**a**), and the experimental quantitation of subcellular localization of FOXO3 is shown in (**b**). Graph represents three independent experiments and error bars represent standard deviations from the mean. ** *p* < 0.001.


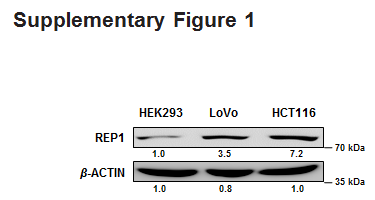


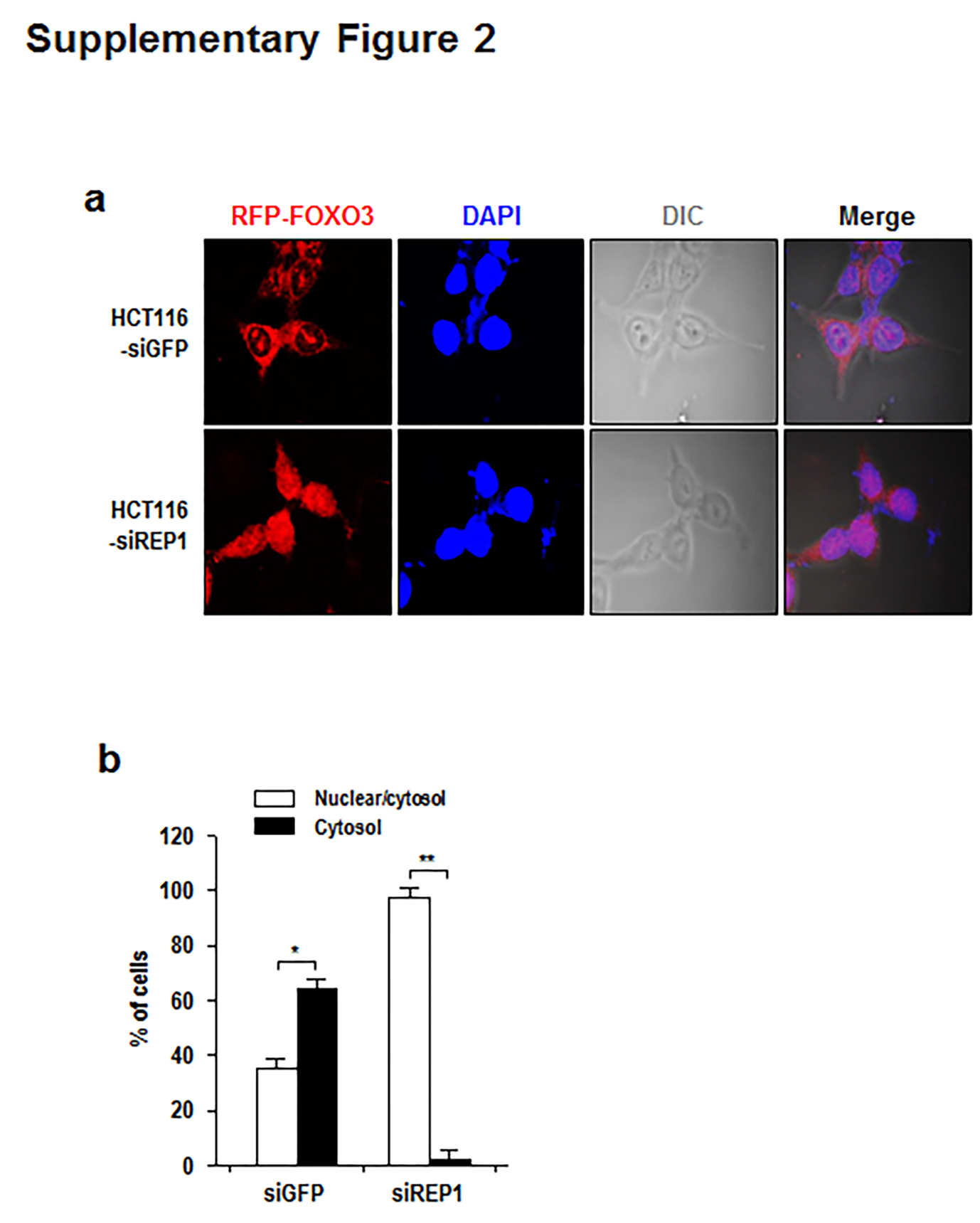

Supplement: Supplementary Information [file cddis2016462x1.docx]
